# Annual variation in predation risk is related to the direction of selection for brain size in the wild

**DOI:** 10.1038/s41598-019-48153-w

**Published:** 2019-08-14

**Authors:** Kim Jaatinen, Anders P. Møller, Markus Öst

**Affiliations:** 1 Nature and Game Management Trust Finland, Degerbyvägen 176, FI-10160 Degerby, Finland; 2 Ecologie Systématique Evolution, Université Paris-Sud, CNRS, AgroParisTech, Université Paris-Saclay, F-91405 Orsay Cedex, France; 3 Environmental and Marine Biology, Faculty of Science and Engineering, Åbo Akademi University, Artillerigatan 6, FI-20520 Turku, Finland; 4 Novia University of Applied Sciences, Raseborgsvägen 9, FI-10600 Ekenäs, Finland

**Keywords:** Behavioural ecology, Evolutionary ecology, Evolutionary theory

## Abstract

The direction of predator-mediated selection on brain size is debated. However, the speed and the accuracy of performing a task cannot be simultaneously maximized. Large-brained individuals may be predisposed to accurate but slow decision-making, beneficial under high predation risk, but costly under low risk. This creates the possibility of temporally fluctuating selection on brain size depending on overall predation risk. We test this idea in nesting wild eider females (*Somateria mollissima*), in which head volume is tightly linked to brain mass (*r*^2^ = 0.73). We determined how female relative head volume relates to survival, and characterized the seasonal timing of predation. Previous work suggests that relatively large-brained and small-brained females make slow versus fast nest-site decisions, respectively, and that predation events occur seasonally earlier when predation is severe. Large-brained, late-breeding females may therefore have higher survival during high-predation years, but lower survival during safe years, assuming that predation disproportionately affects late breeders in such years. Relatively large-headed females outsurvived smaller-headed females during dangerous years, whereas the opposite was true in safer years. Predation events occurred relatively later during safe years. Fluctuations in the direction of survival selection on relative brain size may therefore arise due to brain-size dependent breeding phenology.

## Introduction

A common explanation for the evolution of relative brain size is a trade-off between energetic constraints and natural selection for enhanced cognitive abilities^1^. Under this scenario, predation has been proposed to be a major force shaping cognitive abilities and brain size^2^. This is because the threat of predation may select for greater investment into neural tissues that help animals evade predators, hence resulting in a positive association between (relative) brain size and predation pressure^3^.

However, recent studies have challenged the view that predation should select for larger brains, instead finding the opposite pattern^4,5^, or no relationship at all^6^. Taken together, these findings suggest that the ecological and behavioural context in which predators and prey interact may largely determine how the brains of prey species evolve in response to predators. They also emphasize the paradox that direct evidence for the action of natural selection on cognitive traits is rare^7^, despite the alleged benefits of cognitive ability, especially in the context of predator avoidance.

The shortage of evidence for evolutionary change in brain size in the wild, as opposed to laboratory settings^7,8^, suggests that the selective landscape in natural populations may be more complex than currently appreciated. First, the possibility of temporally fluctuating selection is routinely overlooked when investigating how predators may affect the cognitive evolution of their prey. Temporal fluctuations in the strength and direction of selection may slow down evolution, both over geological and contemporary timescales^9,10^, although the importance of fluctuating selection for evolutionary dynamics on contemporary timescales remains poorly understood^11^. Nevertheless, Losos *et al*.^12^ demonstrated that a dramatic predation-induced reversal in the direction of natural selection is possible even within a single generation.

Second, evolution of a large brain incurs fitness costs other than physiological ones, which have so far received the bulk of attention^13–15^. A pervasive constraint on decision-making performance is that the speed and the accuracy of performing a task cannot be simultaneously maximized (‘speed-accuracy trade-off’)^16^. Animals making fast decisions act to maximize short-term gains because they may lack the cognitive ability to gain as much from additional sampling^17^. In contrast, slow decision-makers take time to make more accurate decisions. Hence, large-brained individuals may occupy positions more at the slow/accurate end of the speed-accuracy continuum, and vice versa. Another implication is that neither speed alone nor accuracy alone may be adaptive, so that a range of different problem solving strategies may yield similar fitness payoffs^17^.

In the context of predator avoidance, the cost of making erroneous decisions increases with predation risk, since errors often carry the ultimate cost of death^18^. Accurate decision-making requires gathering information from an individual’s surroundings, which increases sampling time^6^, with potential repercussions on many aspects of fitness, such as foraging efficiency^19^. The benefits of such slow but accurate decision-making may only accrue under challenging situations, i.e., when predation pressure is high. Correspondingly, slow decision-making may be detrimental during benign conditions, e.g. when predation pressure is relaxed^20,21^. This is particularly true if the reward of the decision decreases with time spent pondering, as is the case with the fitness value of broods or litters with advancing timing of breeding^22^.

Here we explore how relative brain size (i.e., absolute brain size accounting for body size) relates to survival of wild, individually marked female eider ducks (*Somateria mollissima*) exposed to naturally varying predation pressure during incubation. In this ground-nester with female-only incubation, annual predation rates on adult females peak during the incubation period, resulting in considerably lower survival of adult females than males in populations subject to high predation risk, such as ours in the northern Baltic Sea^23^.

In our study population, relatively large-headed females breed later and have higher nesting success during years of high predation risk, whereas females with small relative head size nest earlier and exhibit higher nesting success in low-predation years with early breeding phenology^20^. This finding is consistent with recent evidence from studies of nest predation in waterfowl suggesting that severe predation pressure is associated with a rapid increase in predator occurrence at the beginning of the breeding season (i.e., the predator occurrence probability curve is concave), whereas a slower increase in the probability of predator occurrence which then accelerates over time (i.e., a convex curvature) is coupled with lower overall losses due to predation^24^. Because predation risks on incubating females and nests are positively correlated^25^, we therefore hypothesised that females with relatively larger brains have relatively higher survival during years with high risk of predation, whereas small-brained females may do better during benign years, assuming that predation then disproportionately affects late-breeding, large-brained individuals. We compared the seasonal timing of predation among years differing in overall predation pressure to test the hypothesis that predation rates would rapidly increase and then level off in years with high level of danger, whereas safe years would be characterized by an increase in predation rates towards the end of the breeding season. If found to be present, such seasonal dynamics in predation pressure may expose late-breeding, large-brained females to a higher risk of depredation during safe years.

## Results

### Female survival in relation to relative brain size

**Figure 1:**
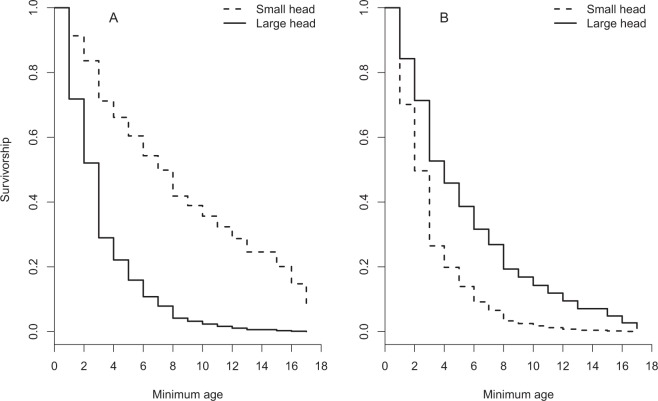
Survivorship of female eiders with increasing age is related to both head size and annual risk of predation (killed females per nesting attempt). In benign years with low predation risk (panel A), small-headed females have higher survival, whereas the opposite is true during years of severe predation pressure (panel B). To illuminate the differences, the figures illustrate low and high extremes with respect to observed annual predation risk (see Methods). For head size, extreme values (referred to as ‘small’ and ‘large’ brains) represent the 10% and 90% quantiles, respectively.

The proportional hazards model explained 10.3% of the variation in female survival. Survivorship was related to annual predation risk on females (b = 0.13 ± 0.16 SE), absolute head size (b = 0.14 ± 0.16 SE), and an interaction between annual predation risk and absolute head size (b = −0.32 ± 0.16, z = −2.06, p = 0.0399), whereas the forced covariate structural size (radius-ulna length) was not associated with survivorship (Table 1). This significant interaction arose because female survival showed a positive relationship with head size in dangerous years, while the relationship was negative in safe years (Fig. 1A,B).

**Table 1:** Model selection table for a proportional hazards model explaining survival of female eiders.

Variable	b	exp(b)	se(b)	z	p
**Relative head volume**	**0**.**14**	**1**.**15**	**0**.**16**	**0**.**87**	**0**.**39**
**Radius-ulna length**	**−0**.**19**	**0**.**83**	**0**.**18**	**−1**.**04**	**0**.**30**
**Annual predation risk**	**0**.**13**	**1**.**14**	**0**.**16**	**0**.**78**	**0**.**44**
**Relative head volume:annual predation risk**	**−0**.**32**	**0**.**73**	**0**.**16**	**−2**.**06**	** < 0**.**04**
Condition index	−0.13	0.88	0.19	−0.69	0.49
Relative head volume:condition index	0.30	1.35	0.26	1.17	0.24
Year of death	0.43	1.54	0.50	0.86	0.39
Condition index:annual predation risk	0.20	1.22	0.20	0.98	0.33

Covariates in bold font constitute the final model.

### Seasonal timing of predation in relation to overall annual predation risk

**Figure 2:**
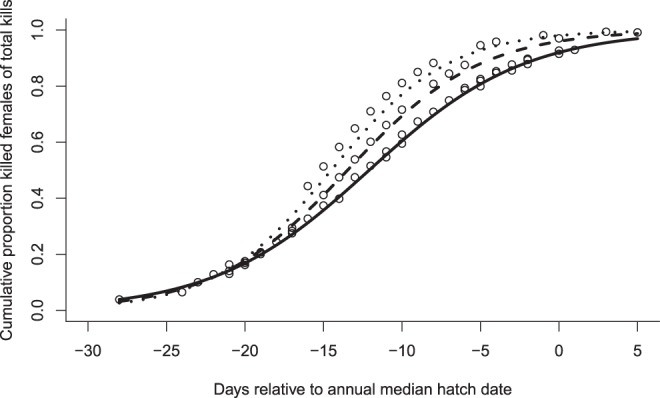
The cumulative proportion of killed female eiders of the sum of annual kills is explained by an interaction between adult predation risk (killed females per nesting attempt) and time relative to the annual median hatch date. In more dangerous years, the cumulative proportion of killed females increases more steeply during the early breeding season. The effect of relative timing within the breeding season on the cumulative proportion of kills is shown at high (+1 SD, dotted line), mean (dashed line), and low (−1 SD, solid line) levels of annual predation risk (see Methods).

The logistic GLMM of the annual cumulative proportion of killed females of total kills over the season provided an adequate fit to the data (marginal R^2^ = 0.478, conditional R^2^ = 0.48). This cumulative proportion was explained by annual predation risk (b = 13.37 ± 2.69 SE, *z* = 4.97, p < 0.001), time relative to the median annual hatch date (b = 0.18 ± 0.92 SE, z = 7.99, p < 0.001), and an interaction between annual predation risk and time relative to the median hatch date (b = 0.61 ± 0.18 SE, z = 3.47, p = 0.0005). The interaction revealed that in years with a higher level of danger, the increase in the cumulative proportion of females being killed was more pronounced during the early breeding season (Fig. 2).

## Discussion

Our results point at an unacknowledged mechanism that may contribute to explaining the inconsistent relationship between brain size and predation under natural conditions^4,5,26^. Thus, brain-size specific breeding phenologies may, in the face of variable predation risk, give rise to temporally fluctuating selection pressure on brain size. While underappreciated in the field of cognitive ecology, the notion that fluctuating selection may maintain animal personality variation in the wild is widely accepted^27^. Studies pinpointing the ecological driver of variation in selection are still scarce, however^12,28^. Here, we showed that there was a temporal shift towards later occurrence of predation events on nesting females in years characterised by relaxed predation pressure (Fig. 2). This should place large-brained, late-breeding females at a disadvantage during years with low overall predation pressure (‘benign conditions’), an effect that was confirmed by our survival analysis (Fig. 1A). We believe that the seasonal difference in the timing of predation may be strong enough to explain a substantial part of the interactive relationship between relative head volume and annual predation risk on survivorship. Thus, based on mean predicted hatching dates (see Fig. 3 in ref.^20^), females having the largest head volumes in our study population breed on average ca 8 days later than their counterparts with the smallest head volumes, a considerable time difference corresponding to ca 30% of the typical 26-day incubation period in the species.

The result that large-brained females enjoyed a survival advantage under high predation risk is expected under the cognitive buffer hypothesis^29^. This is because a greater number of potential attacks and/or a greater diversity of predator species utilizing diverse hunting tactics might demand higher behavioural flexibility^30^. However, our results stand out from previous work in one key aspect. In our case, the greater versus lower odds of survival of large-brained females during years of high and low predation, respectively, may be a passive outcome of brain-size specific differences in breeding phenology. Thus, the mechanism presented here may be more parsimonious: it does not rest on the stringent assumption that large-brained individuals possess superior abilities to hide or flee from predators, nor does it require active preference for smaller-brained prey by predators^31^. Instead, female eiders may simply place different premiums on ‘fast’ versus ‘slow’ decision-making during nest-site selection depending on their relative brain size, as suggested by our previous work^20,21^. Our present results add further support to the notion that neither cognitive strategy consistently outperforms the other; the outcome is context-dependent. Although negative fitness effects associated with greater sensitivity to perceived predation hazard by large-brained individuals have seldom been reported, our finding is nevertheless not unique. Thus, Cole *et al*.^32^ report a parallel example: problem-solving great tits *Parus major*, presumably more sensitive to disturbance during breeding, showed an increased rate of nest desertion with detrimental effects on fitness.

Because the patterns of fluctuating selection on brain size observed here may also be the result of selection on correlated traits, we need to evaluate alternative explanations. The design of our statistical analysis ruled out a correlation between brain and body size. Another recently highlighted correlation may be that between brain size and boldness^17,33^. In our study population, we have collected long-term data (2008-) on boldness of incubating females, quantified as flight initiation distance (FID), i.e., the distance at which females take flight when approached by a possible predator (here, a researcher) in a standardized fashion^34^. FID shows high inter-annual repeatability within females^34^, but also depends on nest cover^35^. However, any confounding between relative brain size and boldness is unlikely in this case, because there is no relationship between relative head volume and FID, controlling for proportional nest cover (see Supplementary Table 1). Finally, the fact that the recovered female eiders were depredated rather than scavenged *post mortem* makes it less likely that energetic trade-offs between neural investment and other fitness-relevant traits such as immunity would explain the patterns observed here. Nevertheless, the amount of variation explained by our survivorship model was relatively modest (*r*^2^ = 0.103). Although small to intermediate effect sizes like those reported here are the most common in biology^36^, such effects can be very important across a few generations.

Interestingly, fluctuating selection on phenotypic traits seldom translates into fluctuating evolution on contemporary time scales^11^. One common explanation is that fluctuating selection will only act to slow down the rate of adaptive evolution if the direction of selection changes regularly and the mean selection differential equals zero^10,37^. These assumptions are unlikely to hold also in the present case. First, the absolute numbers of killed females is considerably higher in dangerous than in safe years (correlation between annual predation index and absolute number of killed females in 1998–2016: r = 0.90, n = 19, p < 0.001). Hence, the difference between the trait mean (here, relative brain size) of the population before and after an annual bout of selection is likely to differ between safe and dangerous years. Second, the study population is currently facing continuously increasing predation pressure on incubating females^25^. ‘Safe’ years, allowing for potential reversals of the positive trend between relative brain size and survival, may therefore occur increasingly rarely. Given the limited annual variation in present-day predation pressure, a longer time frame of research may be helpful. A particularly fruitful avenue of future research may therefore be to contrast the brain-size distribution of the contemporary eider population from the Baltic Sea with that from the period preceding the phase of rapid increase in eagle and mink *Mustela vison* populations, and here museum specimens of eiders may prove useful.

To conclude, our study may provide new insights into the conundrum of why exposure to predators does not necessarily result in the evolution of larger brains in the wild. Thus, the direction of survival selection on relative brain size of eider females fluctuated annually depending on prevailing predation pressure. We argued that brain size-dependent breeding phenology may provide a plausible mechanism for the variable association between relative brain size and survival. Brain size-dependent breeding phenologies may stem from differential emphasis on speed versus accuracy in selecting nest sites^20,21^. Our results suggest that neither fast nor slow decision-making is consistently superior over the other. As a next step, we encourage studies investigating whether brain-size specific breeding phenologies are a general phenomenon, and, if so, the fitness consequences of such divergent nest-site selection strategies should be evaluated under variable predation risk. Elucidating the role of fluctuating selection in maintaining individual variation in brain size will critically depend on the availability of individual-based long-term data.

## Material and Methods

### Study site and fieldwork methods

Handling of birds was carried out in accordance with the Finnish Act on Animal Experimentation. Handling procedures were approved by the National Animal Experiment Board (Permit numbers HY-85-2003, ESLH-2009-02969/Ym-23, ESAVI/1697/04.10.03/2012, ESAVI/2831/04.10.07/2015) and complied with the regulations of Tvärminne Zoological Station.

This study was conducted at Tvärminne (59°50′N, 23°15′E), western Gulf of Finland, during 1998–2016. We used three data sets. First, our focal data set consisted of dead females for which we had complete breeding data and head volume measurements (n = 47). These females were ringed during 1998-2015 and found killed close to their nests during 2012-2016 (head size measurements began in 2012). Second, we determined female body condition for all trapped individuals in 1998–2016 (n = 3228, range: 111–249 females annually). Inclusion of female body condition was motivated by the potential importance of body condition as a predictor of survival in female eiders^38^. Third, we documented annual adult predation risk by recovering all incubating females killed at their nests, regardless of ringing status, and related this number to the total number of nesting attempts annually. These data were also used to determine the cumulative seasonal frequency distribution of dates of recovery of the killed females. The annual adult predation risk and seasonal timing of deaths were determined for the period 2012-2016 (n = 211 killed females, range 16–74 annually) to match the period when the killed females with complete data on head size and other attributes were found (first data set, see above).

Female eiders were captured with hand nets during the end of incubation to minimize nest desertion. Upon capture females were weighed (to the nearest 5 g), measured for structural size (length of the radius-ulna to the nearest 1 mm) and ringed with a standard metal ring. Head width, length and height to the nearest 0.01 mm was measured in 2012–2016. Head volume (cm^3^) was approximated as an ellipsoid using the following equation:

$$4/3\times {\rm{\pi }}\times ({\rm{head}}\,{\rm{length}}-{\rm{beak}}\,{\rm{length}})/2\times ({\rm{head}}\,{\rm{width}})/2\times ({\rm{head}}\,{\rm{height}})/2$$

Although head volume was measured in 2012-2016, we assume, based on high within-individual repeatability of this measure (0.86)^21^, that this estimate of head volume also represents the individual’s past head volume. By doing so, we extended the available data set to years from which we have breeding biology data but lack annual head volume data^21^.

Based on incubation stage, we estimated female body condition for all trapped individuals in 1998–2016, provided that they had incubated their eggs for >8 days (egg laying may otherwise still be in progress)^39^. This was done by regressing the log-transformed projected weight at hatching against the log-transformed radius-ulna length and using the standardized residuals as a condition index (data from all years were pooled to obtain a global index). The projected weight at hatching was obtained by subtracting the expected weight loss (by calculating the expected number of days before hatching, based on incubation stage) from the body weight measured at capture. We caught females at different incubation stages, which enabled us to quantify average weight loss by regressing the logarithm of body weight against the logarithm of incubation time and projected hatching date^39^. The assumption of continued weight loss after female capture is valid in our study population, and thus our index for estimating body condition is reliable^40^.

We documented annual adult predation risk by recovering all incubating females killed at their nests during nest censuses and dividing this number by the total number of nesting attempts in each year^25,41^. The total number of nesting attempts was the sum of actively incubated, depredated and abandoned nests, as well as nests in which the ducklings had already hatched when the nest was first encountered^25^. The recovery dates of the carcasses were used to construct an annual cumulative frequency distribution of the seasonal timing of predation events on nesting females (2012-2016). Although the carcasses were not always fresh when found, we have no reason to expect systematic bias in our sampling of carcasses related to annual predation risk. Thus, the total length of the nest monitoring season (mean ± SD = 21.6 ± 3.36 days) did not vary significantly depending on annual predation risk, as defined above (r = −0.18, n = 5, p = 0.78). Our nest visits were also done at a phenologically equivalent time with respect to annual predation risk: the time difference between the median date of nest visits and annual median hatching date (mean ± SD = −11.84 ± 1.33 days) was not significantly related to annual predation risk (r = 0.23, n = 5, p = 0.72). Furthermore, the order of visiting the study islands remained roughly the same throughout the study period.

### Validating the head size-brain size relationship

We measured head volume for 15 fresh eider females collected in Denmark in 2015. The brains were subsequently extracted from the skull and weighed on a Sartorius precision balance to the nearest 0.001 g. Brain mass could be predicted by head volume according to the following linear regression equation:

Brain mass = 5.37 (SE = 0.27) + 0.199 (SE = 0.03) × Head volume, F = 39.21, d.f. = 1, 13, r^2^ = 0.73, p < 0.0001.

One individual with an extreme brain mass and head volume had a Cook’s D of 0.75, which is still less than the standard criterion of D > 1 for excluding outliers. Log-transformed brain mass and log-transformed head volume produced a model with r^2^ = 0.75.

### Statistical analyses

To test the effects of relative brain size on survivorship, we constructed a proportional hazards model^42^. In this model, survival is modelled as a function of an unspecified baseline hazard at time t, which is modified by the explanatory variables (Table 1) included in the model:

$$h(t,\,{x}_{1},\,{x}_{2},\,{x}_{3},\ldots )={h}_{0}(t)\times \text{exp}({b}_{1}\times {x}_{1}(t)+{b}_{2}\times {x}_{2}+{b}_{3}\times {x}_{3}+\ldots )$$

with h_0_ being the baseline hazard; x_1_ a time-dependent explanatory variable; x_2_, x_3_ explanatory variables; and b_1_, b_2_, b_3_ the corresponding coefficients. In the full model, survivorship was explained by absolute head volume (continuous variable), length of the radius-ulna, annual predation risk (continuous variable), mean body condition index, and year of death. We also included the two-way interactions between head volume and annual predation risk, head volume and mean body condition, and mean body condition and annual predation risk. Using a mean body condition index, i.e., the mean of all observations for each female during 1998–2016, was justified because annual body condition of breeding eider females shows considerable individual repeatability among years^38,43^. Within-individual repeatability of body condition was 0.46 in the present data (95% CI: 0.41–0.50, p < 0.001, n = 2208 observations on 704 females), estimated using the rptR package^44^ and after eliminating females with only a single observation over time.

The proportional hazards model was reduced by sequential removal of nonsignificant variables (α = 0.05), except for radius-ulna length, which was forced into the final model to assess the effects of relative rather than absolute head size on female survival (larger females may be expected to have larger heads and brains). Variables were retained in the final model based on the change in deviance between models fitted with and without each term until only significant terms remained. We maintained the hierarchical structure within our models by including both component main effects in case of finding a significant interaction term^45^. Significant interactions between continuous variables were visualized following the guidelines of Aiken and West^46^. In this procedure, the slope of the independent variable on the dependent variable is estimated at designated levels of the moderating variable. Because of the limited number of years and killed females included in the proportional hazards survival analysis, we computed regression slopes only for observed extreme values of the variables included in the significant interaction (Fig. 1a,b).

Second, we analysed the effect of annual adult predation risk on the seasonal timing of predator-induced mortality events. To this end, we constructed a generalized linear mixed model (GLMM) with binomial error distribution (logit link function) and Laplace parameter estimation, in which the cumulative proportion of females being killed of the sum of annual kills was explained by the adult predation index (continuous variable), time relative to the annual median hatch date, and their two-way interaction. The time difference in days between the focal date and median hatch date (repeated measure), nested within year, was included as a random effect. This random effect allowed us to account for the non-independence of consecutive points on the cumulative frequency curve of killed females over the course of the season. In this analysis, significant interaction terms between continuous variables were graphically illustrated by estimating the slope of the independent variable on the dependent variable at high (+1 SD), mean, and low (−1 SD) levels of the moderator^46^ (Fig. 2). Marginal and conditional R^2^ values were obtained using the function ‘r.squaredGLMM’ provided in the package ‘MuMIn’^47^.

All analyses were conducted using the software R 3.0.2.^48^.

## Supplementary information

Supplementary material

Data 1

Data 2

## Data Availability

All data used for the analyses are available from the corresponding author on reasonable request.
