# Supplementary material for: Annual variation in predation risk is related to the direction of selection for brain size in the wild
Source: Sci Rep. 2019 Aug 14;9:11847. doi: 10.1038/s41598-019-48153-w (PMC6694153; doi:10.1038/s41598-019-48153-w)
Supplement: Supplementary file 1 — Supplementary material [file 41598_2019_48153_MOESM1_ESM.docx]

**Scientific Reports - Electronic Supplementary Material**

**Title: Annual variation in predation risk is related to the direction of selection for brain size in the wild**

Kim Jaatinen^1*^, Anders P. Møller^2^ and Markus Öst^3,4^

^1^Nature and Game Management Trust Finland, Degerbyvägen 176, FI-10160, Degerby, Finland

^2^ Ecologie Systématique Evolution, Université Paris-Sud, CNRS, AgroParisTech, Université Paris-Saclay, F-91405 Orsay Cedex, France

^3^ Environmental and Marine Biology, Faculty of Science and Engineering, Åbo Akademi University, Artillerigatan 6, FI-20520 Turku, Finland

^4^ Novia University of Applied Sciences, Raseborgsvägen 9, FI-10600, Ekenäs, Finland

*kim@luontojariista.fi

**Table S1.** To control for the possibility that our findings of fluctuating survival probability depending on relative brain size in eider females are not the result of varying selection on boldness, which is potentially correlated with brain size, we constructed a linear mixed model where female-specific mean flight initiation distance (FID), a repeatable measure of boldness, was explained by absolute head volume, structural size (length of radius-ulna) and mean proportional nest cover. In the model island identity was used as a random effect to control for potential non-independence of FIDs among individuals nesting on the same island. The data were collected in 2008-2016 and included observations of 341 females nesting on 27 different islands.

No significant relationship was detected between relative head volume (head volume controlled for structural size) and FID.

Figure S1. The relationship between Head volume and brain mass in eiders.
